# Current evidence of neurological features, diagnosis, and neuropathogenesis associated with COVID-19

**DOI:** 10.1590/0037-8682-0477-2020

**Published:** 2020-10-05

**Authors:** Marzia Puccioni-Sohler, André Rodrigues Poton, Milena Franklin, Samya Jezine da Silva, Rodrigo Brindeiro, Amilcar Tanuri

**Affiliations:** 1 Universidade Federal do Estado do Rio de Janeiro, Escola de Medicina e Cirurgia, Rio de Janeiro, RJ, Brasil.; 2 Universidade Federal do Rio de Janeiro, Programa de Pós-Graduação em Doenças Infecciosas e Parasitárias, Rio de Janeiro, RJ, Brasil.; 3 Universidade Federal do Rio de Janeiro, Instituto de Biologia, Rio de Janeiro, RJ, Brasil.

**Keywords:** SARS-CoV-2, Coronavirus disease, Neuropathogenesis, Nervous system, Cerebrospinal fluid

## Abstract

Recent reports indicate that besides respiratory and systemic symptoms among coronavirus disease (COVID-19) patients, the disease has a wide spectrum of neurological manifestations (encephalitis, meningitis, myelitis, acute disseminated encephalomyelitis, metabolic and acute hemorrhagic necrotizing encephalopathy, cerebrovascular diseases, Guillain-Barré syndrome, polyneuritis cranialis, dysautonomia, and myopathies). The severe acute respiratory syndrome coronavirus 2 (SARS-CoV-2) can spread from the respiratory system to the central nervous system, using transneuronal and hematogenous mechanisms. Although not every COVID-19 patient will test positive for the virus in the cerebrospinal fluid exam, the appearance of neurological symptoms associated with SARS-CoV-2 infection reveals the importance of understanding the neurologic manifestations and capacity for neural invasion associated with the pathogen. These aspects are relevant for correct diagnosis and treatment, and for the potential development of vaccines. This review highlights the latest evidence of SARS-CoV-2 infection with a focus on neurological involvement and potential neuropathogenesis mechanisms.

## INTRODUCTION

Severe acute respiratory syndrome coronavirus 2 (SARS-CoV-2) is the etiological agent of the infectious disease known as coronavirus disease (COVID-19)^1^ ^,^ ^2^. The disease affects the respiratory tract in a characteristic manner. This new viral infection is responsible for a pandemic that has already impacted every country worldwide and that generates concerns due to its high transmission potential and mortality rate, particularly in certain risk groups^1^ ^,^ ^2^. In seven months since the first report, more than 16 million cases have been confirmed by the World Health Organization, with more than 600,000 deaths registered worldwide, and more than 2.5 million cases and 90,000 deaths in Brazil^3^.

The virus belongs to the *Coronaviridae* family (Nidovirales order), made up of viruses enveloped in a positive-sense single strand of RNA. SARS-CoV-2 is the seventh coronavirus known to cause disease in humans, much like two others that also belong to the *Betacoronavirus* genus: severe acute respiratory syndrome coronavirus (SARS-CoV) and Middle East respiratory syndrome-related coronavirus (MERS-CoV), which have already been responsible for epidemics in the past^4^.

Coronaviruses have neuroinvasive and neurotropic properties and cause severe neurological complications such as encephalitis and Guillain-Barré syndrome. The purpose of the present review is to summarize our understanding of neurological disorders associated with COVID-19, bringing current evidence of the potential mechanisms of neurological injury (immune mediated, direct viral damage, hypoxia, and hypercoagulability).

## EPIDEMIOLOGICAL AND CLINICAL FINDINGS

According to a retrospective study that investigated 1,099 patients in China, COVID-19 tends to present the following signs and symptoms, in order or prevalence: fever (88.7%), dry cough (67.8%), fatigue (38.1%), productive cough (33.7%), dyspnea (18.7%), and arthralgia or myalgia (14.8%)^5^. In a series of cases reported by the Chinese Center for Disease Control, of 44,672 patients with confirmed diagnoses of COVID-19, the average mortality rate was 2.3%. However, some factors make certain populational groups present greater mortality rates, such as age (between 70 and 79 years of age the mortality rate is 8.0%; above 80 years of age the death rate is 14.8%), as well as pre-existing comorbidities such as cardiovascular disease (10.5%), diabetes (7.3%), chronic respiratory disease (6.3%), high blood pressure (6.0%) and cancer (5.6%). Patients with no pre-existing comorbidities had a mortality rate of 0.9%^6^. 

## NEUROLOGICAL FEATURES OF SARS-COV-2 INFECTION

A retrospective study of 214 cases of patients with confirmed diagnoses of COVID-19 hospitalized at three hospitals in Wuhan, China, found neurological symptoms in 36.4% of patients^7^. Patients in critical condition were more likely to develop neurological manifestations than those with mild or moderate presentations of the disease (45.5% vs. 30.2%). Neurological involvement was classified according to localization: central nervous system (CNS), peripheral nervous system (PNS), and musculoskeletal. Among the CNS manifestations which occurred in 24.8% of patients, the most common were dizziness (16.8%), headache (13.1%), altered level of consciousness (7.5%), and acute cerebrovascular disease (2.8%), defined as ischemic or hemorrhagic stroke. Among the PNS manifestations, which presented in 8.9% of patients, the most prevalent were hypogeusia (5.6%), hyposmia (5.1%), and neuralgia (2.3%). Musculoskeletal involvement, defined as myalgia (muscle pain) and elevated levels of creatine kinase in the blood (>200 U/L) were found in 10.7% of patients. In addition to this, critically ill patients had disorders of multiple organs, including the liver, kidneys, and muscular damage^7^. Other studies conducted worldwide have confirmed these findings^8^. A European study detected an 85.6% rate of olfactory dysfunction and 88% rate of gustatory dysfunction among 417 patients with mild-to moderate COVID-19, with a 44% recovery rate^9^. Anosmia was the initial symptom in 12% of COVID-19 cases^9^. 

Helms et al.^10^ found neurological disorders in 90% of patients with SARS-CoV-2 infection in France. However, their study had some limitations. Seven of 58 (12%) patients had a history of previous nervous system involvement, and others used sedative medications. Meningitis/encephalitis was reported in a patient with SARS-CoV-2 infection, which brain magnetic resonance imaging revealed to be ventriculitis and encephalitis with predominance in the temporal lobe and hippocampus^11^. Another study detected viral encephalitis as a single manifestation of COVID-19^12^. Viral RNA was found in the cerebrospinal fluid (CSF) in both reports, indicating that SARS-CoV-2 may invade the CNS^11^ ^,^ ^12^. Cases of Guillain-Barré syndrome associated with SARS-CoV-2 have been published^13^. An Italian study showed that three out of five cases were consistent with an axonal variant of Guillain-Barré syndrome, and with a demyelinating process in two others^13^. The diagnosis was based on positive test results of nasopharyngeal samples for SARS-CoV-2 at the onset of the syndrome in four patients, and one had reactive serologic test for the virus. There was an interval of five to ten days between the onset of COVID-19 and the first symptoms. Lower-limb weakness and paresthesia were found in four patients, and facial diplegia, ataxia, and paresthesia in another. All the CSF samples showed negative results on RT-PCR assay for SARS-CoV-2, with normal white blood cell counts (fewer than 5 leukocytes/mm^3^). Three patients had elevated protein levels (>40 mg/dL) in CSF. Electromyography showed fibrillation potentials in four patients (three in the early phase and one on the 12th day)^13^. The mechanisms underlying cerebrovascular disease manifestations in COVID-19 are probably multifactorial (viral, immune, hypoxia, and hypercoagulability). For this reason, there are reports of acute stroke associated with COVID-19 not only in individuals with risk factors, but also in the younger population^7^ ^,^ ^14^. Table 1 summarizes the spectrum of neurological manifestations associated with COVID-19^7^ ^,^ ^15^ ^-^ ^17^. 

**TABLE 1:** Neurologic manifestations of COVID-19 .

Neurological disorders of COVID-19
*Central nervous system diseases*
Encephalitis, meningitis, myelitis, meningoencephalitis
CNS demyelinating disease
Post-infectious acute disseminated encephalomyelitis
Post-infectious brainstem encephalitis
Encephalopathy
Movement disorders
Acute hemorrhagic necrotizing encephalopathy
Cerebrovascular disease: ischemic and hemorrhagic stroke, cerebral venous sinus thrombosis, venous and arterial thromboses, subarachnoid hemorrhage associated with immune thrombocytopenic purpura
*Peripheral nervous system*
Guillain-Barré syndrome
Miller Fisher syndrome
Mononeuropathy
Polyneuritis cranialis
Optic neuritis
Dysautonomia
*Muscle involvement*
Myalgia
Myopathies
Rhabdomyolysis

## LABORATORIAL DIAGNOSIS

Available laboratory tests capable of detecting SARS-CoV-2 are based on molecular biology principles (real-time reverse transcription polymerase chain reaction [RT-PCR]) and immunological testing (specific IgG and IgM antibodies). Viral detection using real-time RT-PCR in respiratory samples from the oropharynx, nasopharynx, sputum, and/or endotracheal aspirate or bronchoalveolar lavage in severe respiratory disease continues to be the preferred method of laboratory testing for the diagnosis of symptomatic patients in the acute phase of the disease (the first week after onset of symptoms)^18^. A negative RT-PCR assay result does not exclude the infection, considering that the sensitivity of RT-PCR is approximately 60%^18^. Moreover, the viral load may be undetectable during the mean incubation period (first five days). After the second week of infectious symptoms of the disease, when patients develop an immune response, immunological tests using blood samples (validated serological tests) are recommended^19^. Neurological disorders caused by SARS-CoV-2 have been mainly reported during the first two weeks after the onset of infectious symptoms^7^. So far, few cases of encephalitis/meningitis with positive RT-PCR assays of the CSF have been described^11^ ^,^ ^12^ ^,^ ^20^. This can be attributed to several reasons: the RT-PCR assay of CSF may yield negative results when performed early; the method has limited sensitivity associated with false negative tests in the CSF; and CNS viral clearance in cases of early neuroinvasion induces a low viral load in the CSF^21^. On the other hand, not all neurological complications of COVID-19 occur due to direct viral action. Systemic viral infection may be the trigger for neurological autoimmune-mediated diseases^22^. Post-infectious and parainfectious nervous system autoimmune diseases, as well as metabolic and cerebrovascular disorders are reported to be associated with COVID-19^13^ ^,^ ^16^ ^,^ ^17^. Under these conditions, the virus may not be detected in the CSF. Therefore, the laboratory results should be evaluated along with the nervous system condition, including immunological and molecular examinations of all samples that detect the presence of the virus in the patient, not only in CSF^11^ ^,^ ^17^. Specific IgM in CSF has been found in cases of negative RT-PCR assays. This marker may be promising for the diagnosis of neuro-COVID-19^23^ ^-^ ^25^.

In addition to analyzing the presence of the virus in the CSF, the inflammatory profile (pleocytosis, high level of protein, blood-CSF barrier dysfunction, and intrathecal synthesis of immunoglobulins) and albuminocytological dissociation (normal cell count and high levels of protein) should be investigated in CSF. Although not every COVID-19 patient will test positive for the virus in the CSF exam, the appearance of neurological symptoms in the course of infection with SARS-CoV-2 indicates that it may still represent a neurologic manifestation associated with infection^13^.

## EVIDENCE OF NEUROPATHOGENESIS

**FIGURE 1:**
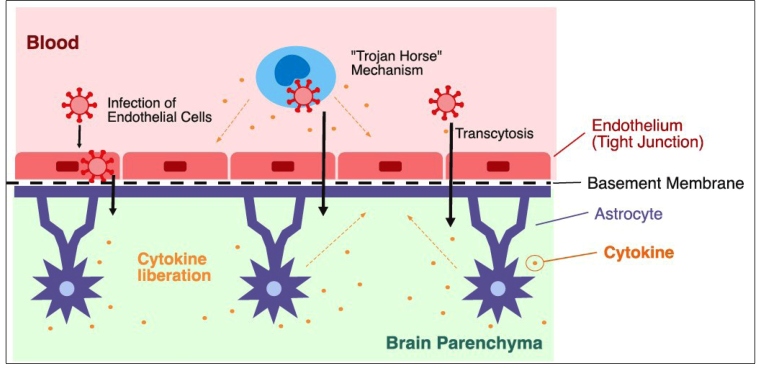
Potential mechanisms of SARS-CoV-2 neuroinvasion and neurovirulence in the central nervous system via the blood-brain barrier (capillary endothelial cells with tight junctions) (Adapted from Puccioni-Sohler & Rosadas, 2015)^43^.

**FIGURE 2:**
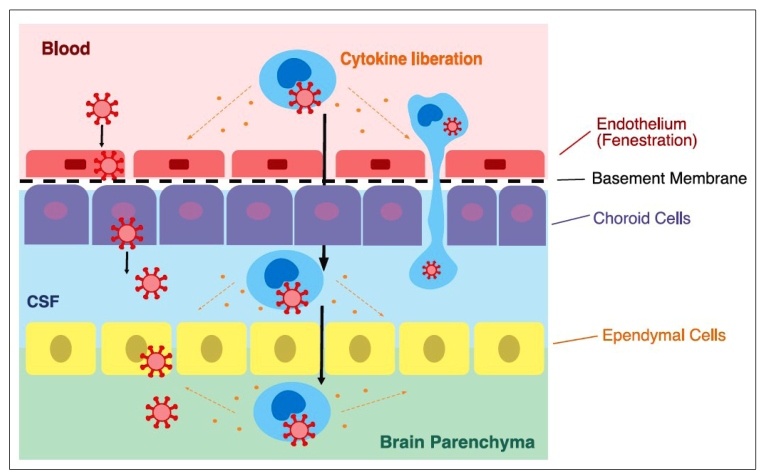
Potential SARS-CoV-2 neuroinvasion and neurovirulence in the central nervous system via the blood-CSF barrier (the choroid plexus represents a barrier in the brain separating the blood from the CSF) (Adapted from Puccioni-Sohler & Rosadas, 2015)^43^.

The mechanism through which the virus affects the nervous system is not entirely clear yet. Some authors have proposed hypotheses based on evidence related to the SARS-CoV-2 and other coronaviruses, particularly SARS-CoV and MERS-CoV^26^ ^,^ ^27^. A possible mechanism is direct damage due to the presence of the virus in the CNS. This could occur by two main pathways. First, via the hematogenous route, which has already been described for other coronaviruses (Figure 1 and Figure 2)^28^. SARS-CoV-2 particles have already been identified in capillary endothelia and neurons of frontal lobe brain sections in an autopsy of a COVID-19 patient, corroborating this hypothesis^29^. Second, via the neuronal route, by which the virus infects nerve endings and migrates through the synapses until it reaches the CNS, which has already been described in SARS-CoV^30^. The olfactory nerve is mainly held responsible for the infection of the CNS through the neuronal pathway due to its proximity to the nasal cavity, a common locus of virus infection, and the communication of the nasal epithelium with the olfactory bulb, through the cribriform plate of the ethmoid bone, thereby allowing the entrance of the virus^28^ ^,^ ^30^. This possibility is supported by the findings of hyposmia or anosmia in the early stages of COVID-19^9^. In addition, brain magnetic resonance imaging showed signal alteration in the olfactory bulbs and frontal lobe of a patient with anosmia with SARS-CoV-2 infection. After disappearance of the symptoms, the control image was also normal^31^.

The functional receptor used by SARS-CoV-2 for cellular invasion is the angiotensin-converting enzyme 2 (ACE2) receptor, expressed in different tissues, including the endothelium of small blood vessels in the brain’s microcirculation, and, to a lesser extent, in cells of the CNS (neurons and glia cells)^32^. The virus infects the cells using the S1 spike protein to interact with the cellular ACE2 receptors^33^. This could explain how the virus overcomes the blood-brain (brain capillaries endothelial cells) and blood-CSF (choroid plexus epithelium of the ventricles) barriers to invade the CNS via the hematogenous route (Figure 1 and Figure 2).

The hypotheses of direct infection of the CNS is also corroborated by the description of patients with COVID-19 from whom the virus was isolated in samples of CSF, which characterizes a condition of viral encephalitis, as well as the case of another patient with neck stiffness, seizures, and lowered consciousness level, who also had SARS-CoV-2 in the CSF, but not on the nasopharyngeal swab, which characterizes viral meningitis^11^ ^,^ ^21^. In addition, the detection and sequence of the virus was demonstrated in the CSF of a patient suspected of clinically isolated syndrome, a CNS demyelinating disorder^34^. 

Other mechanisms that would account for neurological conditions in COVID-19 are hypoxia and immune injury. Hypoxia, caused by pulmonary infections, generates swelling in the brain and intracranial hypertension, impairing blood flow to the brain and leading to acute ischemic stroke. Immune injury, explained by the so-called proinflammatory “cytokine storm” provoked by SARS-CoV-2 infection, can cause damages to brain cells and to the skeletal muscle^24^ ^,^ ^26^ ^,^ ^33^. Activated monocytes in the bloodstream produce proinflammatory cytokines that may damage the blood-brain or blood-CSF barriers, oligodendrocytes, neurons, and muscles (Figure 1 and Figure 2)^26^. 

Another mechanism that has been suggested is based on the fact that ACE2 participates in the regulatory mechanisms both of blood pressure and of blood flow to the brain, with reduced expression in patients previously suffering from high blood pressure^34^ ^-^ ^36^. With SARS-CoV-2 infection, this function becomes even more impaired, leading to hypertensive peaks that may result in brain hemorrhage^37^. In parallel, patients with COVID-19 present elevated levels of D-dimer and antiphospholipid antibody^38^, which may be related to thrombotic phenomena and can increase the probability of cerebrovascular diseases^39^.

## FINAL REMARKS

Infections of the nervous system affect populations worldwide and have high rates of morbidity, mortality, and long-term sequelae, which are associated with emerging and reemerging viruses, such as some arboviruses and enteroviruses, as well as co-infection with other endemic diseases^40^ ^,^ ^41^. Cases of SARS-CoV-2 infection with invasion of the nervous system are being described with the emergence of the new pandemic due to the infectious disease COVID-19. In addition to the clinical definitions of nervous system infections, there is a need to identify these emerging pathogens and their neuroinvasive mechanism for various purposes, such as the production of new vaccines, evaluation of migration and possible mutations, epidemiological control in each region of the world, and the diagnosis of the neurological disease itself. Patients with COVID-19 may present initially with neurological symptoms or experience neurological complications during hospitalization. The diagnosis of neuro-COVID-19 can be challenging. In endemic areas, healthcare professionals should be aware of the most frequent neurological alterations in COVID-19 patients such as smell and taste disturbances, tinnitus, headache, altered level of consciousness, seizures, and delirium. In addition to the neurological manifestations initially described such as encephalitis, meningitis, Guillain-Barré syndrome, and cerebrovascular diseases, cases of acute disseminated encephalomyelitis, acute hemorrhagic necrotizing encephalopathy, Miller-Fisher syndrome, mononeuropathy, polyneuritis cranialis, optic neuritis, and dysautonomia have also been reported^15^ ^-^ ^17^. Some neurological complications such as stroke, encephalitis, and encephalopathy may contribute to the rapid clinical deterioration and high mortality rate. Respiratory insufficiency may also be associated with viral brainstem encephalitis. The RT-PCR assay for SARS-CoV-2 is still an essential criterion and the gold standard for confirming the diagnosis, despite limited sensitivity. It should be performed not only on CSF samples, but also on oropharyngeal, nasopharyngeal, and bronchial lavage samples, as well as to identify the presence of specific IgM in serum and CSF. The CSF should be examined to confirm SARS-CoV-2 infection, and to exclude other infectious diseases^40^ ^-^ ^42^. 

Since SARS-CoV-2 has neuroinvasive and neurotropic properties, early recognition can help initiate treatment and isolation early, to prevent clinical worsening and spread of the virus and the underreporting of neurological cases associated with COVID-19.
